# Supplementary material for: Extracellular Vesicles Released by Enterovirus-Infected EndoC-βH1 Cells Mediate Non-Lytic Viral Spread
Source: Microorganisms. 2020 Nov 8;8(11):1753. doi: 10.3390/microorganisms8111753 (PMC7695210; doi:10.3390/microorganisms8111753)
Supplement: Supplementary file 1 [file microorganisms-08-01753-s001.pdf]

microorganisms

MDPI

Supplementary Materials

Extracellular Vesicles Released by Enterovirus-Infected EndoC-βH1 Cells Mediate Non-Lytic Viral Spread

Eitan Netanyah [1], Matteo Calafatti [1], Jeanette Arvastsson [1], Eduardo Cabrera-Rode [2], Corrado M. Cilio [1*] and Luis Sarmiento [1*]

[1] Department of Clinical Sciences, Immunovirology Unit, Skåne University Hospital, Lund University, 221 85 Lund, Sweden; eitan.netanyah@med.lu.se (E.N.); matteocalafatti@gmail.com (M.C.); jeanette.arvastsson@med.lu.se (J.A.)

[2] Department of Immunology, National Institute of Endocrinology, Vedado 10400, Havana, Cuba; diabetes@infomed.sld.cu and eduardo.cabrerarode@gmail.com

* Correspondence: corrado.cilio@med.lu.se (C.M.C.); luis.sarmiento-perez@med.lu.se (L.S.); Tel.: +46-704330338 (C.M.C.), +46-70-306-76-26 (L.S.)

Received: 9 October 2020; Accepted: 6 November 2020; Published: date

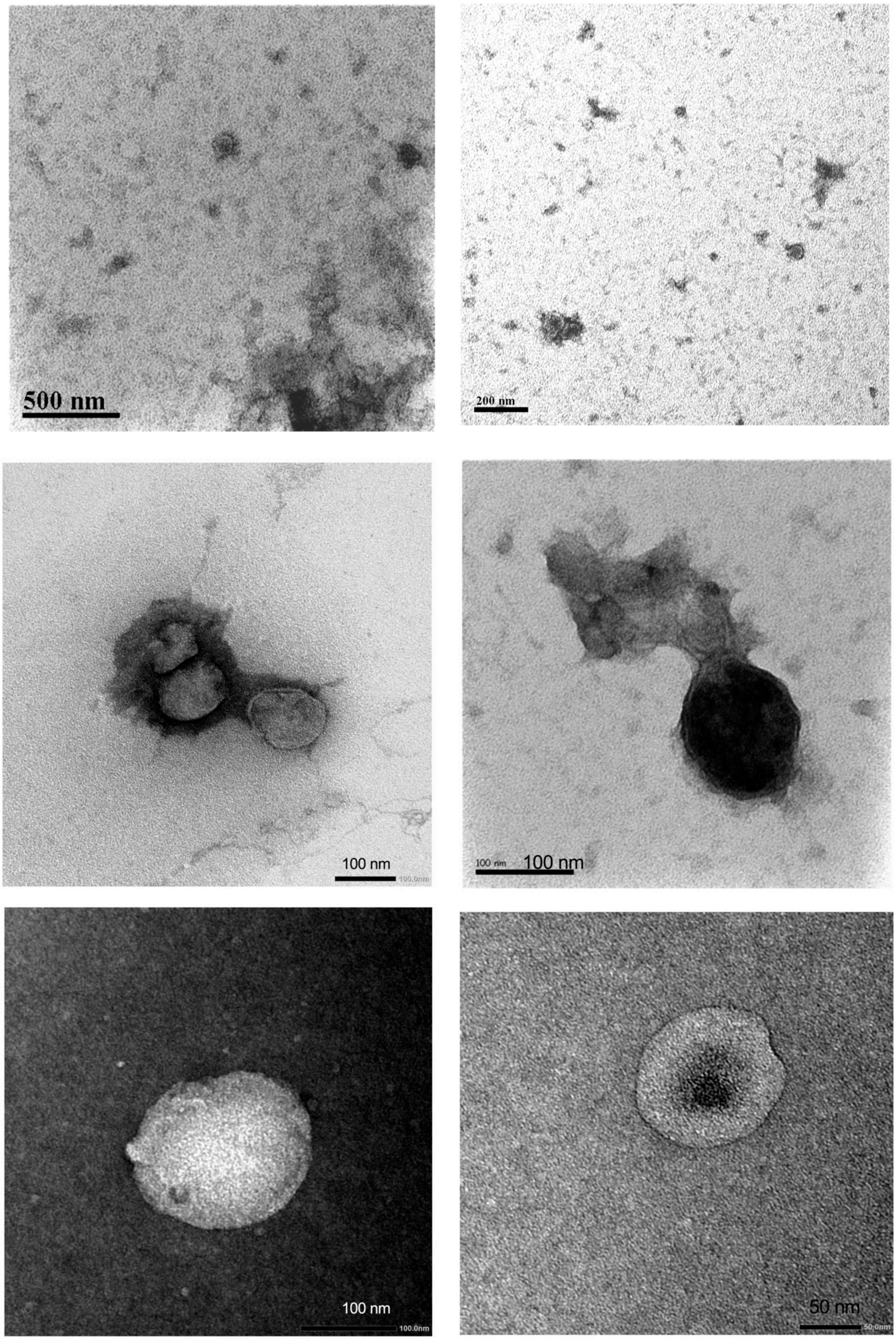

Figure S1 (related to Figure 1). Images of EVs preparations under an electron microscope showing the varying sizes and morphologies of EVs. The images were taken after negative staining by using different magnifications.

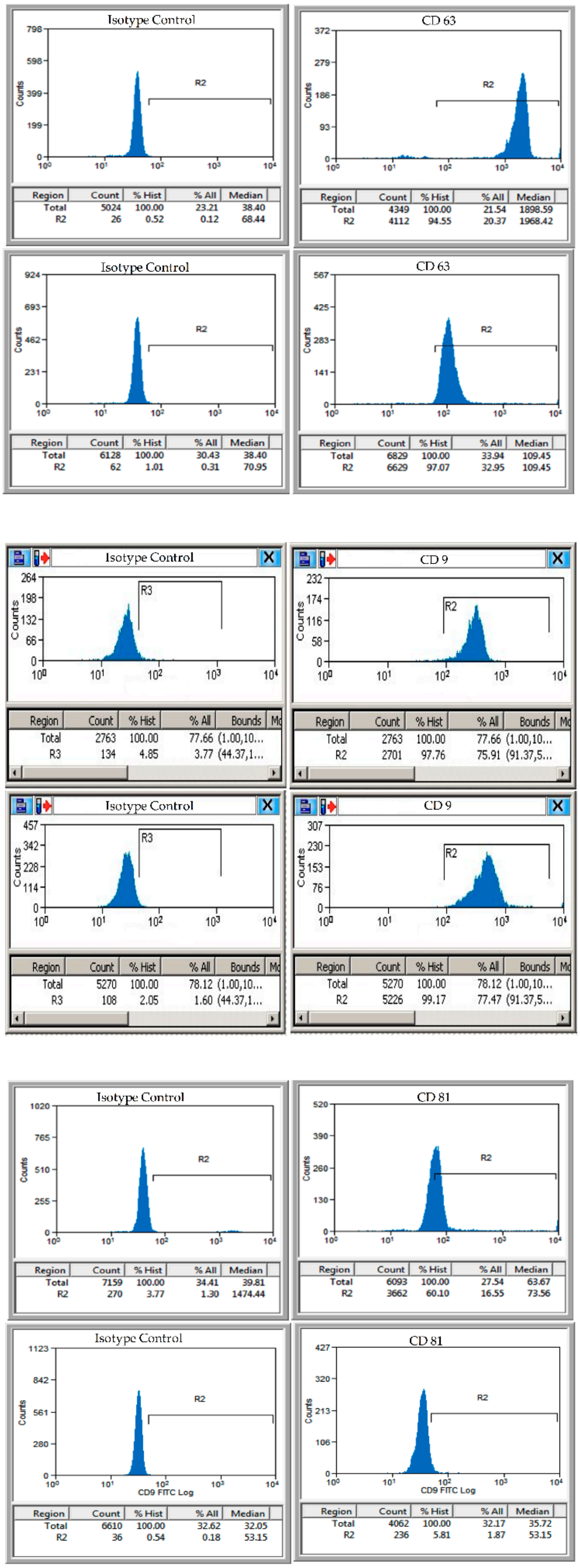

Figure S2. (related to Figure 3A). Flow cytometry detection of EVs captured onto anti-CD63 coated beads. The EVs-bead complexes immunostained against EVs-associated proteins CD9, CD63, and CD81 and compared with the appropriate isotype control.

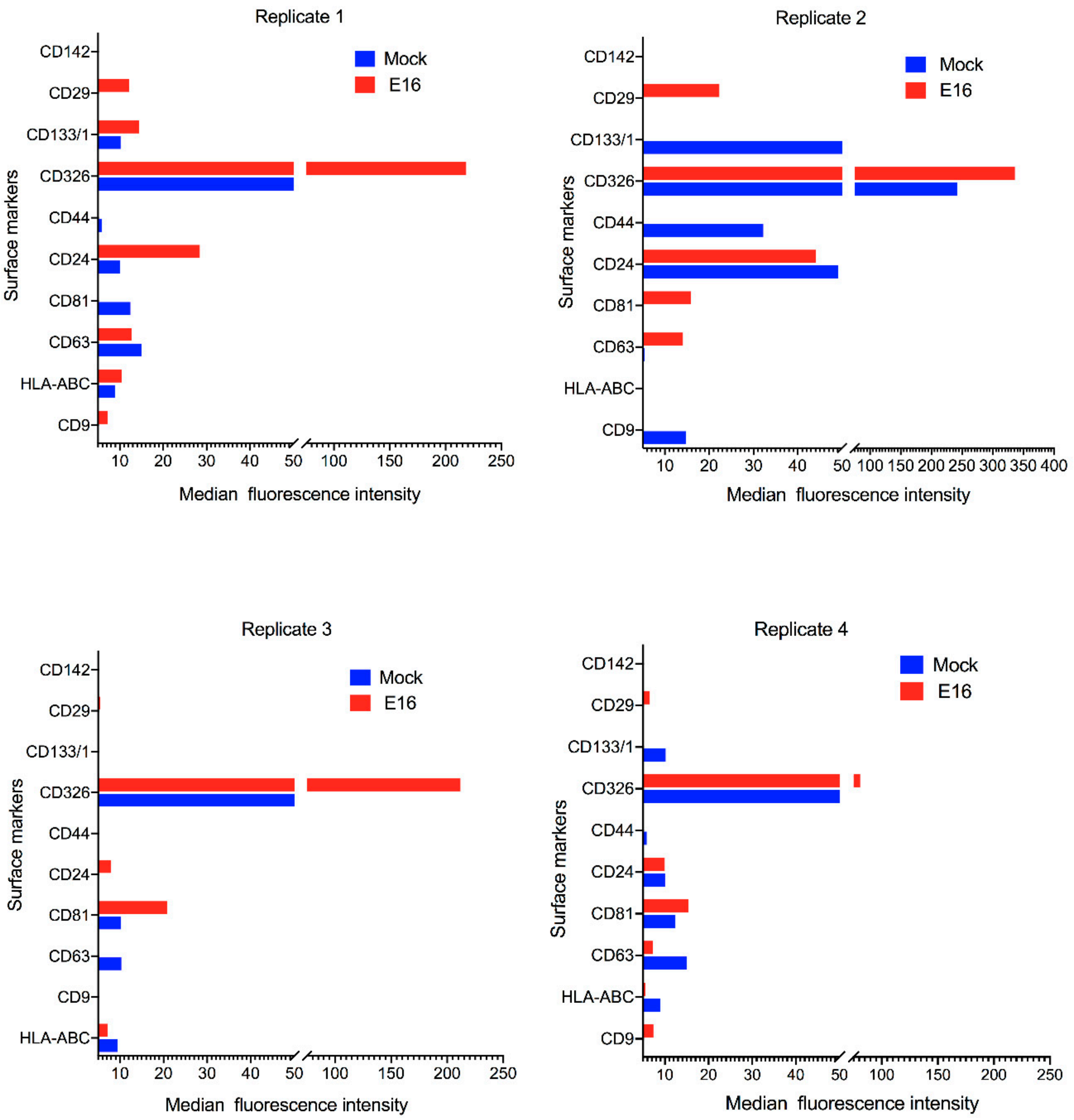

Figure S3. (related to Figure 3B). Median fluorescence intensities values of selected markers detected in EVs isolated from mock and E16-infected EndoC-βH1 cells. Complete surface profiles data set of the results shown in Figure 3B.

Table S1. Calculation of the endpoint neutralization titer by the Reed and Muench method.

Endpoint Neutralization titer	No. of wells on test plate		Accumulated Value		Neutralization	
	+ for NT (w/o CPE)	− for NT (w/o CPE)	+ for NT[a] (w/o CPE)	− for NT[b] (w/o CPE)	Ratio	%
1:8	3	0	20	0	20/20	100
1:32	3	0	17	0	17/17	100
1:128	3	0	14	0	14/14	100
1:512	3	0	11	0	11/11	100
1:2048	3	0	8	0	8/8	100
1:8192	3	0	5	0	5/5	100[c]
1:32768	2	1	2	1	2/3	66.6[d]
1:131072	0	3	0	4	0/4	0

[a] Sum from the bottom. [b] Sum from the top. [c] Neutralizing antibody titer that will prevent infection of 100% of virus-inoculated cells. [d] Endpoint neutralization titer (50% virus neutralization titer) was calculated to be 1:45232.
